# Trends in the Disease Burden and Risk Factors of Women’s Cancers in China From 1990 to 2019

**DOI:** 10.3389/ijph.2024.1607245

**Published:** 2024-12-04

**Authors:** Wei Ning, Jinnan Liu, Yongbo Lu, Bin Zhu, Wei-Hong Zhang, Ying Mao

**Affiliations:** ^1^ School of Public Policy and Administration, Xi’an Jiaotong University, Xi’an, China; ^2^ International Centre for Reproductive Health, Ghent University, Ghent, Belgium; ^3^ School of Public Health and Emergency Management, Southern University of Science and Technology, Shenzhen, China

**Keywords:** breast cancer, cervical cancer, ovarian cancer, disease burden, time trends, risk factors, China

## Abstract

**Objectives:**

To examine age-specific trends and risk factors in the burden of women’s cancers (WCs) in China from 1990 to 2019 to inform strategies.

**Methods:**

Data were sourced from the Global Burden of Disease 2019 and World Population Prospects 2019. Time trends, age differences, and key factors for breast, cervical, and ovarian cancers (BC, CC, and OC) were analyzed based on age-standardized incidence rate (ASIR) and disability-adjusted life years (DALYs) rate.

**Results:**

ASIRs for BC and CC increased over the study period, with a slower growth rate for CC after 2005, likely due to targeted HPV prevention. OC showed the highest ASIR and DALY increases, indicating a growing concern. Peak ASIR for BC and CC was in women aged 50–55, while OC showed a higher burden in women aged 70–79. Lower DALYs in women born after 1985 suggest improved healthcare access.

**Conclusion:**

This study highlights significant trends in cancer burden among Chinese women, driven by age and reproductive health policies. Future efforts should enhance screening, health literacy, and age-targeted risk reduction for specific cancer types.

## Introduction

Worldwide, cancer is the leading cause of premature death and disability [1]. This is particularly evident in women whose physiological structures differ from those of men and exhibit a higher susceptibility to developing cancers affecting the reproductive system or breast tissue [2, 3]. More than three million women are diagnosed with WCs globally per year [4]. In 2018, WCs accounted for 38.6% of new cases and 26.9% of cancer-related deaths among women worldwide [5]. Among WCs, breast, cervical, and ovarian cancers are the three most common, causing huge psychological and economic burdens, especially in low-income and middle-income countries. Breast cancer (BC) is the most prevalent and lethal cancer in women, as reported by the World Health Organization (WHO); 2.3 million new cases of BC and 685 thousand associated deaths were reported worldwide in 2020 [6]. Cervical cancer (CC) is by far the most prevalent cancer affecting the genitals in women. CC is the fourth most common malignant tumor in women, with 604,127 cases and 341,831 deaths worldwide in 2020 [7]. Globally, 313,959 new cases of ovarian cancer (OC) and 207,252 deaths from ovarian cancer (OC) were reported in 2020, making it the eighth most common cancer in women [7]. Although relatively infrequent, ovarian cancer is regarded the most lethal type of female genital cancer.

WCs also pose a serious threat to the wellbeing and health of women in China. China has more than 1/5 of the world’s female population [8], contributing to the large number of WC cases. In 2020, China contributes to over 17% of the global incidence and deaths attributed to BC, CC, or OC [7]. Furthermore, the incidence rates of these WCs in China are increasing, and they have been ranked among the top 10 cancers in Chinese women, which has emerged as a significant public health concern in China. Consequently, gaining a comprehensive understanding of the epidemiological patterns of WCs in China is imperative to facilitate pertinent health policies to guide preventive measures and provide appropriate management strategies for women with cancer.

Understanding the trends and underlying factors driving the burden of WCs in China is essential for developing targeted interventions. While BC, CC, and OC have been prioritized under national health policies, their respective incidence and DALYs trends vary significantly due to differences in risk factors, public health initiatives, and demographic shifts. This study aimed to analyze the time trends, age-specific patterns of incidence and DALYs, and key contributing factors for these three WCs in China from 1990 to 2019, providing evidence to inform future prevention, early detection, and management strategies tailored to the needs of Chinese women. Moreover, this study offered insights into the dynamic interplay between socio-economic changes, policy initiatives, and individual behaviors that influence cancer burden in China.

## Methods

### Data Source

Data on three WCs were identified and extracted based on age groups in China from 1990 to 2019 from the global burden of disease 2019(GBD 2019) database (http://ghdx.healthdata.org/gbd-results-tool) [9, 10]. Following data screening strategies were implemented: the measure was selected as “Incidence” and “DALYs,” metric as “Number” and “Rate,” region as “China” and “Global,” gender as “female,” and disease cause as “B.1.14 Breast Cancer,” “B.1.15 Cervical Cancer,” and “B.1.17 Ovarian Cancer” [11]. Eighteen groups of age ranges (from 0 to 14 years, from 15 to 94 years in 5-year intervals, and 95+ years) were included. Some of the analyses did not include the 0–14 age group because no cases from BC or CC were reported in that age group. The general procedures for data collection and processing in the GBD study have been detailed and validated elsewhere [12, 13]. We also extracted the female population data of China by year (1990–2049) and age group (from 15 to 95+ years old in 5-year intervals) from “World Population Prospects 2019” that was issued by the Department of Economics and Social Affairs of the United Nations [14]. This report records the actual and expected population totals of different countries and territories worldwide between 1950 and 2100.

### Statistical Analysis

**FIGURE 1:**
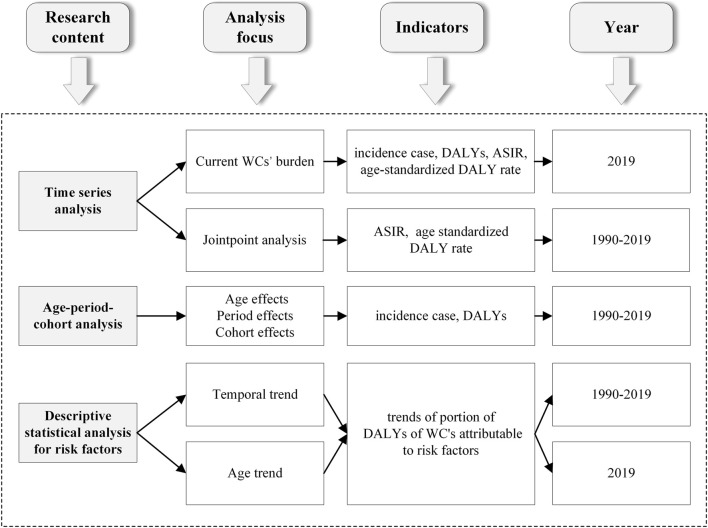
Research framework of this study (China, 1990–2019).

Figure 1 illustrates the research framework of this study. China’s ASIRs and age-standardized DALY rates of BC, CC, and OC were estimated to illustrate the current burden and perform a joinpoint regression analysis of burden trends from 1990 to 2019. Significant trends in age groups were also identified. In joinpoint regression, inflection points are identified in a model to divide the long-term trend in incidence or DALYs of a time series into segments between these points [15]. The annual percentage change (APC) and estimated average annual percentage change (AAPC) generated by regression indicate the magnitude and direction of burden variation, and *p* < 0.05 was considered significant. Furthermore, we conducted age-period-cohort model to calculate the age, period and cohort effects on disease burden of three WCs by using natural logarithm of disease incidence as the dependent variable and selecting median of these datasets as the independent variable. The longitudinal age curve represents the fitted longitudinal age specific rates relative to the reference cohorts adjusted for period deviations. The age effect refers to age-related physiological and pathological changes that affect disease incidence rates. The period rate ratios are the ratios of age-specific rates in a given period compared to the reference period. The period effect refers to changes in disease incidence rate caused by various events over time. The cohort rate ratios are the ratios of age-specific rates in a given cohort compared to the reference cohort. The cohort effects refer to differences in disease bureden between generations as a consequence of lifestyle changes over time or different exposure to risk factors [16, 17]. Local drifts represent the annual percentage change in the expected age-specific rates over time. Net drift represents the annual percentage change in the expected age-adjusted rates over time [18, 19]. The associated important parameters have also been described in more details in the Supplementary 2. The aforementioned analyses include comparisons with global trends.

Additionally, we performed a descriptive analysis of the temporal and age trends of risk factors for each of the three WCs in China. Based on the contribution of risk factors to DALYs for the three WCs reported in the GBD2019, the proportion of DALYs attributable to risk factors (PAF) for each cancer type was calculated to determine the impact and trend of different risk factors.

### Statistic Software

The Joinpoint Regression Program (version 4.9.1.0) was used to analyze trends in ASIR and age-standardized DALY rates for the three WCs during 1990–2019. The parameters for the age-period-cohort models were obtained using the age-period-cohort Web Tool provided by the National Cancer Institute (URL: https://analysistools.cancer.gov/apc/). All figures were plotted using OriginPro software (version 2021).

## Results

### Current Burden in China

Table 1 shows the counts and ASRs of WC disease burden in 2019. For three WCs, China had the largest number of incident cases (368 thousand, 110 thousand, and 46 thousand, respectively), and DALYs (2.88 million, 1.62 million, and 0.84 million, respectively) among all nations in 2019. Although ASRs of cancer burden among Chinese women in 2019 were all lower than the global average, the trends in disease burden indicate a narrowing gap between China and global levels. Moreover, the odds of developing a kind of WC among Chinese women differed substantially by age. The ASIRs and age-standardized DALY rates of three cancers by age group in 2019 showed similar characteristics and trends, peaking at 50–69 years old (Supplementary 1).

**TABLE 1:** The log-transformed joinpoint trends in the disease burden of three women’s cancers (Global Burden of Disease 2019 study, China and the world, 1990–2019).

Location	Cause	Measure	Trend 1		Trend 2		Trend 3		Trend 4		Trend 5		2019 count No.	2019 ASR per 100,000	1990–2019 AAPC (95% CI)
			Years	APC	Years	APC	Years	APC	Years	APC	Years	APC			
China	Breast cancer	Incidence	1990–1996	1.6*	1997–2010	3.2*	2011–2016	1.5*	2017–2019	3.9*	NA	NA	368,375	35.61	2.6* (2.5, 2.7)
		DALYs	1990–1994	−0.2	1995–2001	0.7	2002–2015	−0.8*	2016–2019	1.3*	NA	NA	2,877,240	277.98	−0.2* (−0.3, 0.0)
	Cervical cancer	Incidence	1990–1992	−2.5*	1993–1998	−0.3	1999–2004	5.2*	2005–2019	0.6*	NA	NA	109,760	11.01	1.1* (0.7, 1.4)
		DALYs	1990–1998	−2.2*	1999–2004	4.2*	2005–2009	−2.0*	2010–2017	0	2018–2019	−3.1*	1,622,242	157.5	−0.3* (−0.6, −0.1)
	Ovarian cancer	Incidence	1990–2003	2.4*	2004–2016	1.4*	2017–2019	3.1*	NA	NA	NA	NA	45,482	4.54	2.0* (1.9, 2.1)
		DALYs	1990–2003	1.8*	2004–2016	0.6*	2017–2019	2.8*	NA	NA	NA	NA	835,056	80.52	1.3* (1.2, 1.4)
Global	Breast cancer	Incidence	1990–1994	1.6*	1995–2010	0.3*	2011–2013	−0.1	2014–2019	0.4*	NA	NA	1,977,212	45.86	0.5* (0.3, 0.6)
		DALYs	1990–1994	0.5*	1995–2001	−0.5*	2002–2010	−0.9*	2011–2019	0	NA	NA	20,310,187	473.83	−0.3* (−0.4, −0.3)
	Cervical cancer	Incidence	1990–2003	−0.4*	2004–2012	−0.5*	2013–2016	0.4	2017–2019	−0.5*	NA	NA	565,541	13.35	−0.4* (−0.4, −0.3)
		DALYs	1990–2003	−0.9*	2004–2011	−1.4*	2012–2016	−0.1	2017–2019	−1.0*	NA	NA	8,955,013	210.64	−0.9* (−1.0, −0.8)
	Ovarian cancer	Incidence	1990–1995	0.6*	1996–2002	0.2*	2003–2015	−0.1*	2016–2019	0.8*	NA	NA	294,422	6.87	0.2* (0.1, 0.3)
		DALYs	1990–1995	0.2*	1996–2003	−0.1	2004–2011	−0.3*	2012–2016	0.2	2017–2019	0.8*	5,359,737	124.68	0.0 (0.0, 0.1)

Notes: ASR, Age-standardized rate; APC, annual percentage change; AAPC, average annual percent change; 95% CI, the 95% Confidence interval; NA, Not applicable; *: Significantly different from zero, *P*-value <0.05.

### Long-Term Trends of Burden, 1990–2019

Table 1 also presents the results of the Joinpoint Regression Analysis of burden during 1990–2019. Over 30 years, BC’s ASIR has increased steadily (AAPC = 2.6*, 95% CI: 2.5, 2.7) and dramatically within 1997–2010 (APC = 3.2*) and 2017–2019 (APC = 3.9*). The age-standardized DALY rate was declining from 2002 to 2015 (APC = −0.8*); this trend then reversed since 2016 (APC = 1.3*). However, it presented a slightly downward trend throughout the study period (AAPC = −0.2*, 95% CI: −0.3, 0.0). In general, BC’s ASIR increased faster in China than the corresponding global rates; the age-standardized DALY rate has not shown the same improvement as global. According to the corresponding AAPCs of every age group (Supplementary 1), the ASIR increased at all ages above 15 years; the decline in age-standardized DALY rate was mainly attributed to 35–54 years, but there was still a progressively higher rate across 79–94 years.

Regarding CC, its ASIR, crossed the global level and showed an increasing trend in general (AAPC = 1.1*, 95% CI: 0.7, 1.4). Although ASIR decreased continuously during 1990–1993 (APC = −2.5*) and remained stable until 1998 (APC = −0.3), it increased substantially through 1999–2004 (APC = 5.2*); the growth rate slowed after 2005 but continued to increase annually (APC = 0.6*). The age-standardized DALY rate (AAPC = −0.3*, 95% CI: −0.6, −0.1) declined slightly during 1990–2019. Considering different age groups (Supplementary 1), the increase in the ASIR was most obvious in the age group of 35–44 years; and the decrease in age-standardized DALY rates was mainly manifested in 15–24 years.

Finally, patterns different from the smooth trend of the global burden of OC were observed for ASIR (AAPC = 2.0*, 95% CI: 1.9, 2.1) and age-standardized DALY rate (AAPC = 1.3*, 95% CI: 1.2, 1.4) in China. All increased significantly from 1990 to 2019 and entered a phase of growth spurt during 2017–2019. As for age-specific (Supplementary 1), the substantial increase in ASIR during 1990–2019 occurred across almost all age groups (15–94 years), while the age-standardized DALY rate declined considerably under 19 years, whereas the mid-to-late adulthood (over 45 years) were characterized by increasing age-standardized DALY rate.

### Effects Based on Age-Period-Cohort Model

**FIGURE 2:**
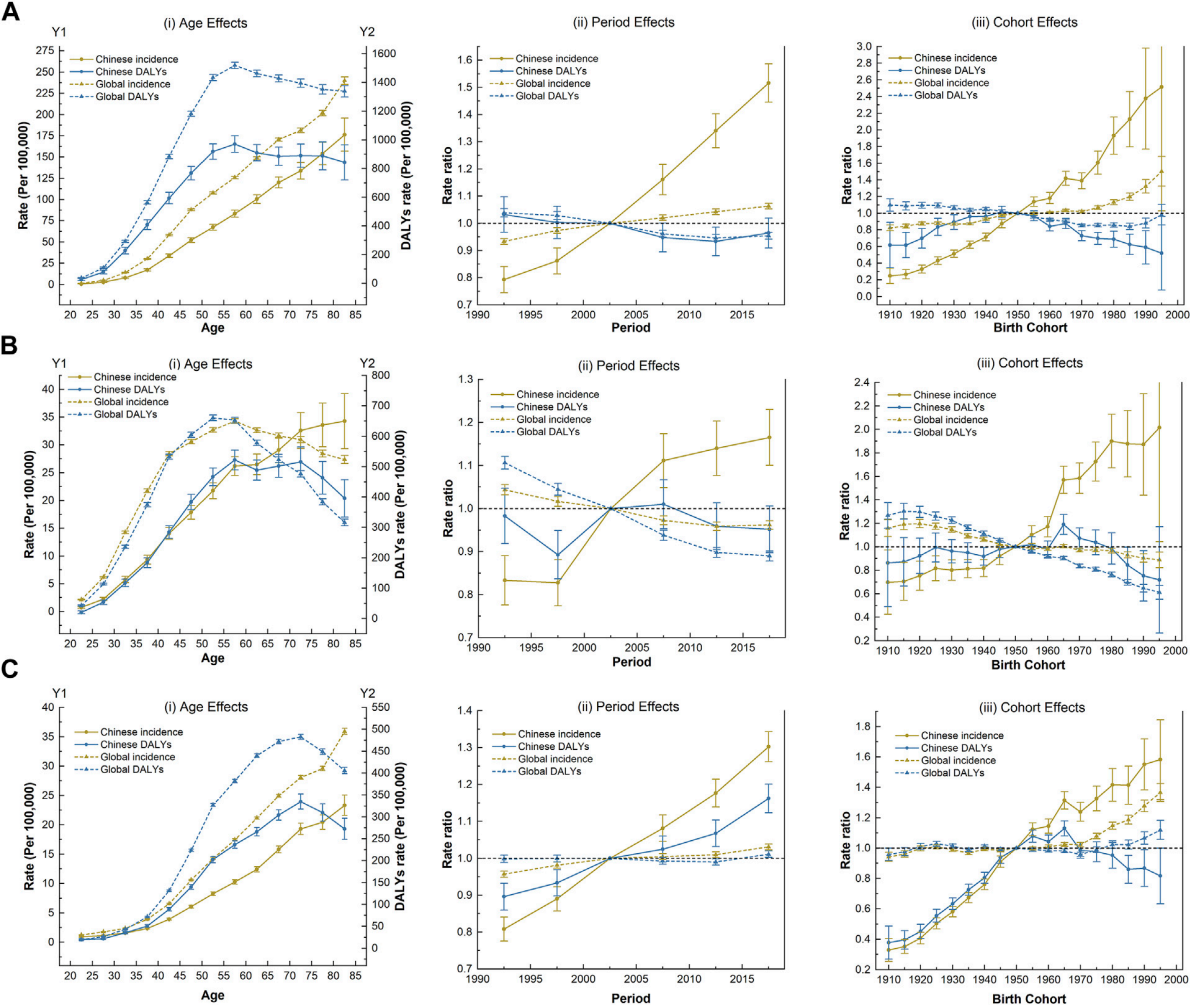
The age-period-cohort effect on the disease burden of three women’s cancers (Global Burden of Disease 2019 study, China and the world, 1990–2019). **(A)** Breast cancer. **(B)** Cervical cancer. **(C)** Ovarian cancer.

Figure 2 shows the estimated age, period, and cohort effects on the disease burden of three WCs. The age, period and cohort effect were statistically significant, and the detailed results were shown in Supplementary 1. Age effects showed that the incidence of the three WCs increased significantly with age in the same birth cohort in China. However, the age effect peaked at DALYs of 55–60 years for BC and CC (70–75 years for OC). Remarkably, the risks from age effects on burden of CC in China exceeded global levels for the same birth cohort after 70 years old (Figures 2Ai, Bi, Ci).

The incidence of the three WCs in China climbed faster than the global level under the period effect and continued to rise over time. Notably, the DALYs of OC has increased considerably. However, China’s success in reducing the DALYs of BC occurred between 2005 and 2015. Favorable period trends in DALYs of CC was also observed over the past decade, but with only a moderate improvement that was not as pronounced as that happened globally (Figures 2Aii, Bii, Cii).

Regarding cohort effects, the incidence risk of three WCs in China generally increased for successive cohorts, especially after the reference cohort (the 1950 birth cohort). The DALYs of three WCs showed almost identical trends with fluctuating variation curves. In the earlier or later birth cohort, the risk of DALYs due to three WCs was low, with respective peaks in the 1945 (for BC) and 1965 cohorts (for CC and OC); among them, the DALYs of OC fluctuated the most with the birth cohort (Figures 2Aiii, Biii, Ciii).

### Attributable Risk Factors of Three Women’s Cancers

**FIGURE 3:**
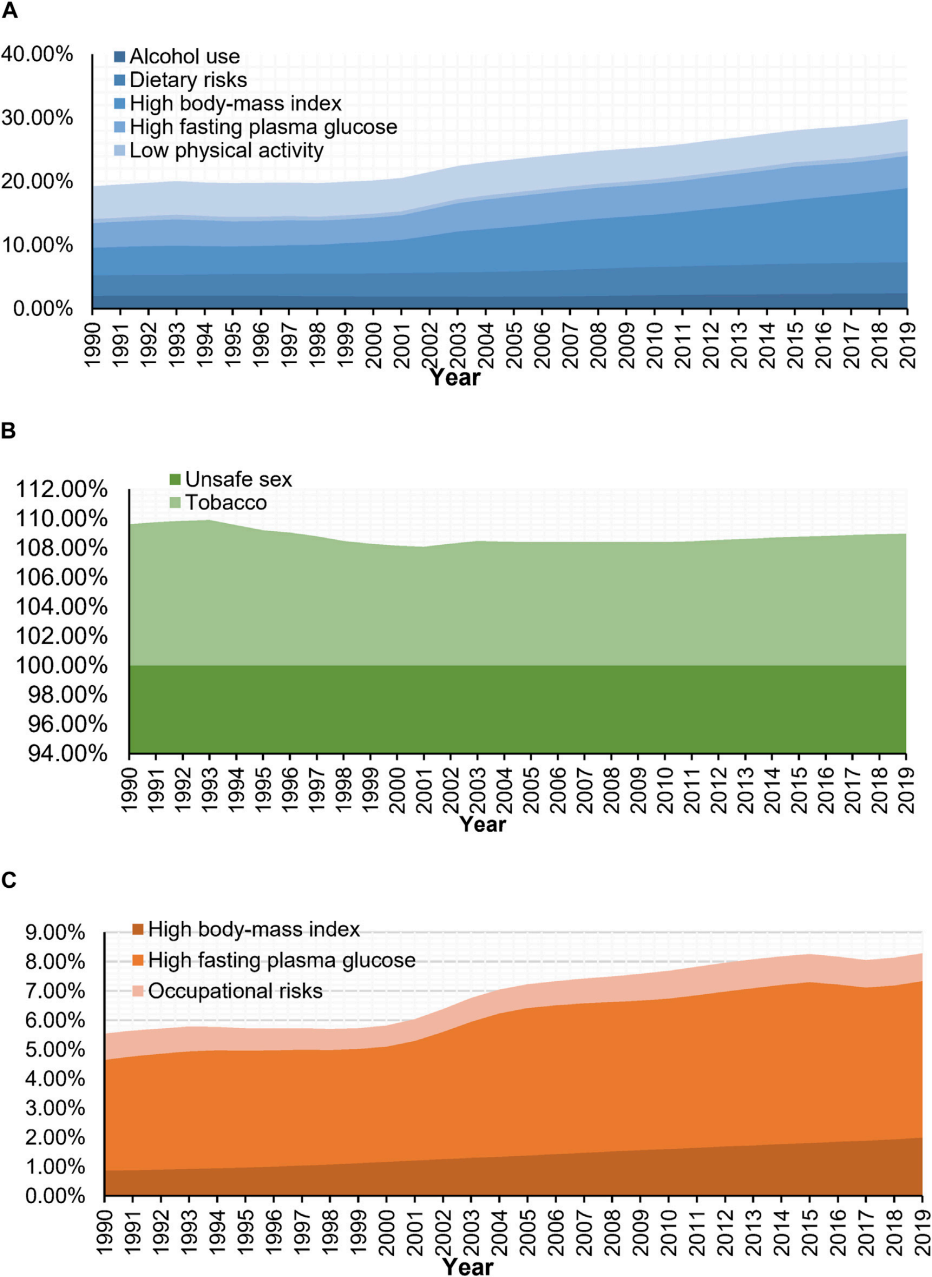
Trends of portion of disability-adjusted life years of women’s cancers attributable to risk factors (Global Burden of Disease 2019 study, China, 1990–2019). **(A)** Breast cancer. **(B)** Cervical cancer. **(C)** Ovarian cancer.

**FIGURE 4:**
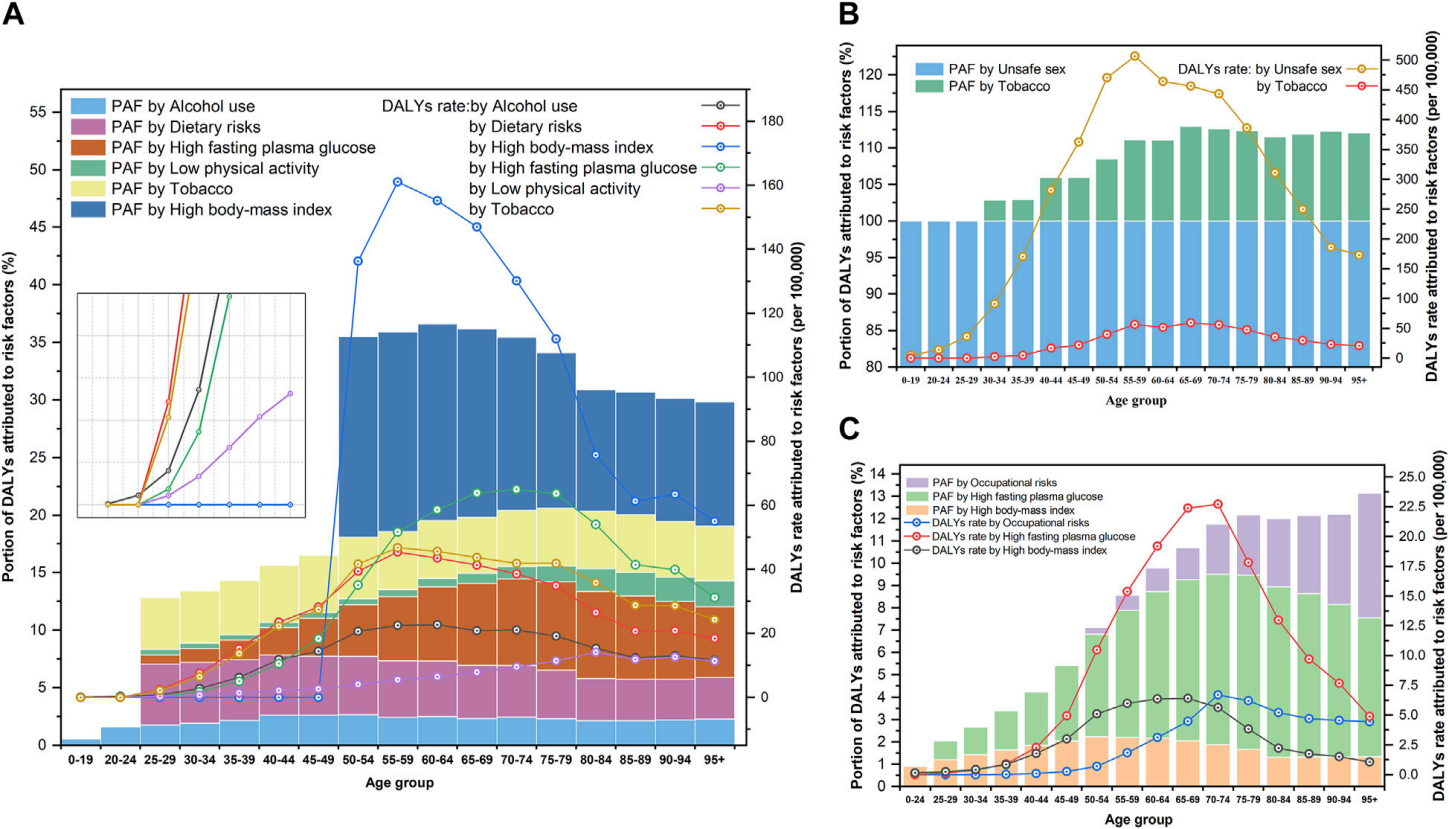
Portion of disability-adjusted life years of women’s cancers and risk factors attributable to disability-adjusted life years rate for age groups (Global Burden of Disease 2019 study, China, 2019). **(A)** Breast cancer. **(B)** Cervical cancer. **(C)** Ovarian cancer.

Of the DALYs due to WCs in 2019, there were six main risk factors for BC, two for CC, and three for OC in the GBD 2019 study. Owing to differences in lifestyle and health literacy, the risk factors in different periods and among age groups varied widely. Figures 3, 4, respectively show the variation in the proportion of DALYs attributable to the corresponding risk factors by year and age for each cancer. Figure 4 also depicts the fluctuation in DALY rate contributable to these risk factors by age in 2019. Detailed results are shown in Supplementary 3.

Based on the DALYs associated with BC in 2019, six risk factors, including 11.66% (95% UI: 3.63, 23.11) attributable to high BMI, 5.10% (95% UI: 0.94, 11.57) attributable to high fasting plasma glucose, 5.00% (95% UI: 1.97, 7.78) attributable to tobacco use, 4.84% (95%UI: 2.32,6.49) attributable to dietary risks, 2.44% (95% UI: 1.77, 3.18) attributable to alcohol use, and 0.73% (95% UI: 0.39, 1.36) attributable to low physical activity, were identified. As for the proportion of DALYs related to CC, it was estimated that 100.00% (95% UI: 100.00, 100.00) was attributed to unsafe sex and 8.98% (95% UI: 3.99, 15.73) was attributed to tobacco use. Moreover, high fasting plasma glucose (the proportion of DALYs attributable to risk factors (PAF = 5.35%, 95% UI: 1.03, 12.75), high BMI (PAF = 1.99%, 95% UI: 0.00, 5.20), and occupational risks (PAF = 0.94%, 95% UI: 0.38, 1.65) were identified as major risk factors for DALYs in OC (Figure 3).

Throughout the study period, high BMI (PAF in 1990: 4.38% to PAF in 2019: 11.66%) and high fasting plasma glucose (3.86%–5.10%) outranked tobacco use (5.14%–5.00%) as the major contributors to DALYs related to BC and were also consistently the main risk factors of DALYs for OC during this period (high fasting plasma glucose: 3.78%–5.35%; high BMI: 0.86%–1.99%). Meanwhile, DALYs associated with CC can almost always be attributed to unsafe sex (100.00%–100.00%) at any time or age group (Figure 3).

Additionally, we observed age-specific differences in the manifestations of risk factors. In 2019, high BMI was the primary risk factor (with an average PAF > 10.00%) for DALYs associated with BC in age groups over 50 years, which negatively correlated with age but did not contribute to DALYs in age groups under 50 years. The PAF of high fasting plasma glucose levels peaked at the ages of 75–79 years (PAF = 7.68%) and subsequently declined with age. Meanwhile, dietary risk, as the third major contributor to the DALYs associated with BC, was the most noteworthy factor in the age group of 25–49 years. For CC, in addition to being the dominant causative factor of unsafe sex, the PAF of tobacco use increased with age but stabilized after 65 years (PAF fluctuated between 11.50% and 13.00%). Regarding high fasting plasma glucose levels as the primary cause of DALYs in OC, exposure to this risk factor should be of increasing concern as age advances. The PAF of occupational risk was proportional to the age in groups older than 50 years (Figure 4).

## Discussion

In this study, we systematically revealed the current burden, risk factors, and long-term trends in incidence and DALY rates by age, period, and birth cohort for three WCs in China. We found that the ASIRs and age-standardized DALY rates for three WCs were lower than the corresponding global levels during 1990–2019. Nevertheless, the AAPC results also revealed adverse trends in China over the past three decades compared with the global trend, indicating that the burden in China accounts for an increasing proportion of the global burden of WCs. Specifically, the ASIR of these three cancers has skyrocketed over the past three decades in China, in contrast to the global pattern of a marginal rise (for BC and OC) or a decline (for CC). In addition, the improvement in age-standardized DALY rates for BC and CC was less pronounced compared to the global trend, while the values for OC showed a significant increase. In the age–period–cohort model, the disease burden of WCs was strongly related to age, period, and birth cohort. We are committed to further exploring the key characteristics and underlying triggers of the trends for three WCs, combining relevant risk factors and China’s context, to support the optimization of prevention and treatment strategies for WCs in China.

### Breast Cancer

The time trend in the ASIR for BC among Chinese women, which slowed in growth from 2011 to 2016 before accelerating again post-2017, presented an intriguing phenomenon likely shaped by multiple socio-economic and policy-related factors. Initially, the rapid increase in ASIR before 2011 can be attributed to the impact of China’s 2009 healthcare reform, which expanded access to essential health services and introduced the Basic Public Health Services program. This policy improved the accessibility of primary health services, resulting in broader routine health management and earlier disease detection, including cancer. Furthermore, the Chinese government also initiated a free breast cancer screening policy in 2009 as part of these healthcare reforms [15, 20]. Consequently, the influx of newly diagnosed cases led to a temporary surge in ASIR. However, the growth rate of ASIR slowed between 2011 and 2016 as the initial wave of diagnoses reached a saturation point, and further screening uptake was limited by a range of challenges [20–23]. Early screening programs, although introduced nationwide, faced issues such as a lack of adequate medical equipment, insufficient outreach, and significant geographical and social disparities in healthcare access [24, 25]. Cultural factors, including the stigma around cancer screening [26], especially in conservative rural areas, further hindered widespread participation, resulting in low overall screening coverage. These limitations led to a moderation in ASIR growth as new cases stabilized after the initial surge. The surge in ASIR after 2017 aligns with the launch of the Healthy China 2030 initiative in 2016, which aimed to further expand cancer prevention efforts, increase health literacy, and encourage public participation in health programs [27]. Public and governmental increasingly focused on early cancer screening efforts, particularly in rural areas with enhanced resources and infrastructure. The combination of expanded screening, improved health literacy, and increased public awareness likely contributed to the post-2017 resurgence in ASIR, as more cases were detected through expanded outreach and participation.

This study revealed significant age-related trends in the BC burden among Chinese women, characterized by shifts in peak age groups for incidence and DALYs. Specifically, the ASIR and age-standardized DALYs rate for BC among Chinese women currently peak between ages 50–54, which aligns with findings from previous studies [28, 29]. Studies have shown that, on average, Chinese women develop BC at least a decade earlier than their Western counterparts [28, 30, 31]. However, we observed a postponement trend: the ASIR of Chinese women aged 55–59 in 2019 was comparable to that of those aged 50–54. This shift may be influenced by aging-related factors. Additionally, Chinese women of reproductive age (25–34 years) experienced a relatively low but dramatic increase in BC ASIR over the past three decades, possibly driven by shifts in lifestyle and reproductive patterns. With socioeconomic advancement [24] and westernization of lifestyles [32], women in this age group often prioritize careers over family life. High work pressure, increased age at first live birth, one-child, and decreased breastfeeding duration have become common characteristics among those [32, 33]. Similar to Western countries, factors such as negative emotions, lower fertility rates, delayed first childbirth, and shorter breastfeeding duration are strongly associated with an increased ASIR of BC [34–36]. These findings emphasized the need for further research on age-specific risk factors and a potential reevaluation of early screening age coverage. The recent adjustment in China’s 2022 Work Plan for Breast Cancer Screening [37] to include women aged 35–64 is a positive step, but as BC burdens shift in an aging population, future adjustments may be necessary to ensure comprehensive coverage of at-risk age groups.

High BMI, high fasting plasma glucose levels, tobacco use, dietary risks, alcohol use, and low physical activity were identified as risk factors for BC. Unexpectedly, we found that high BMI did not increase the risk of BC in premenopausal women (<50–54 age group). Some studies even suggest that being overweight or obese before menopause may have a protective effect against BC development [38–40]. However, this protective effect of high BMI against BC in premenopausal women is less certain and requires further research. In contrast, high BMI is associated with an increased risk of BC in postmenopausal women. Statistically, the rates of overweight and obesity among adults increased from 16.4% to 3.6% in 1992, respectively, to 34.3% and 16.4% in 2019, respectively [41], which correlated with the increasing ASIR of China, approaching that of the world. More than half of the adult population in China is overweight or obese, and the rates of overweight and obesity are virtually identical in male and female [41]. With the westernization of lifestyle (increased consumption of high-sugar and high-fat foods and less physical exercise), the rates of overweight and obesity will further increase in China [32, 42, 43]. By 2030, it is estimated that 354.55 million Chinese adult women will become overweight or obese [44]. This indicates that the risk of developing BC due to high BMI worsens over time. Moreover, high fasting plasma glucose levels increase the burden of BC and have received little attention in China. International evidence has argued that patients with diabetes have a worse prognosis for BC than do non-diabetic patients [45, 46] and that patients with diabetes have an increased risk of developing BC [47, 48].

### Cervical Cancer

We observed an intriguing divergence in the burden trends of CC and BC in China. Both cancers were designated as priority areas for prevention and control at the same time. However, despite being subject to the same cancer control policies, CC transitioned to a period of slower growth much earlier than BC. From 1999 to 2004, the ASIR of CC rose sharply, contributing to a significant increase in age-standardized DALYs rate. After 2005, however, the pace of ASIR growth slowed, and the age-standardized DALYs rate began to decline. We attribute this shift to China’s targeted strategies for sexually transmitted infections (STIs) prevention and HPV exposure reduction, which had a particularly strong effect on CC risk. Specifically, the rapid increase in CC ASIR from 1999 to 2004 likely resulted from rising HPV infection rates, exacerbated by changes in sexual behavior and limited awareness of safe sexual practices. As China urbanized, shifts in sexual health behaviors contributed to increased HPV transmission. At the time, public health infrastructure and HPV-related knowledge were insufficient, and preventive measures such as the HPV vaccine were not yet widely available [49]. In 2005, however, the trajectory of CC ASIR began to change. Unlike BC, whose ASIR continued to increase steadily, CC growth decelerated. This shift coincided with the establishment of a comprehensive STIs prevention framework. Recognizing the importance of sexual health in HPV exposure, China launched extensive public health initiatives to reduce STIs transmission and promote safe sexual practices. The Outline of Cancer Prevention and Control Program in China (2004–2010) identified CC as a priority cancer, enabling the implementation of interventions focused on reducing HPV-related risks, particularly in rural areas [50]. These efforts were further strengthened by the 2006 Regulation on the Prevention and Treatment of HIV/AIDS [51], which mandated health education, free condom distribution, and expanded public health campaigns on safe sex practices. The regulation institutionalized STIs prevention as a key public health issue, establishing mechanisms for local accountability and interdepartmental coordination across various levels of government. These interventions significantly impacted HPV transmission rates, leading to early stabilization in CC ASIR as HPV-related risks were mitigated. In contrast, BC, though also prioritized by health policies, did not benefit from the same direct risk reduction measures related to its primary risk factors. Therefore, CC’s unique trajectory under the same policy framework highlights the effectiveness of disease-specific risk management strategies, particularly in reducing HPV-related exposures that drive CC risk. Our findings underscore the need for similarly targeted interventions to address lifestyle and environmental risk factors for other cancers prevalent among women.

Age and birth cohort affected the CC burden. The ASIR increased the fastest in women aged 30–44 years, and later birth cohorts were more likely to develop CC. Sexual attitudes, promiscuity, and HPV and other STIs may be to blame [52]. Central Asia, Europe, and Japan also saw rising ASIR among young women [53, 54]; thus, this phenomenon was not unique to China. Conversely, the age-standardized DALY rates of each age group did not increase, with women aged 15–24 showing the greatest decline. Women born after 1985 had a lower risk of DALYs. This population exhibited better economic development and had better healthcare, insurance, and social conditions. In 2019, the ASIR peaked at 50–54 years of age, 5 years earlier than that in 1990. The age-standardized DALYs rate peaked between 50 and 54 years of age.

This study found that unsafe sex and tobacco use contributed to the CC disease burden. Unsafe sex is a common absolute risk factor for CC. According to epidemiological evidence, factors such as early sexual activity, multiple sexual partners, and oral contraceptives, which can transmit HPV and STIs, play a role in the etiology of CC [55, 56]. However, with the availability of HPV vaccines, HPV infection in young women can be prevented early, thereby reducing the incidence of CC. China approved bivalent, quadrivalent, and nine-valent HPV vaccines during 2016–2018 [57, 58], a decade after their approval in developed countries [59]. Widespread HPV vaccination in China is warranted as an effective primary preventive measure. Nevertheless, China’s vaccine coverage is hindered by affordability [60], accessibility [61, 62], and lack of awareness [63–65]. Vaccine shortages [61] and unequal distribution of healthcare resources between urban and rural areas [66] caused poor availability and spatial accessibility. The cost of the imported HPV vaccine is often prohibitive for many young women, especially those in rural and low-income areas [67, 68]. Misconceptions about the side effects of the vaccine and insufficient knowledge of its safety and efficacy are barriers to vaccination. Moreover, smoking has been shown to increase the risk of CC by affecting the cervical cells and weakening the immune system [69].

### Ovarian Cancer

Our study found that the ASIR and age-standardized DALYs rate of OC have both increased over the past 30 years, with a noticeable rise after 2017. This trend indicated that OC has been an escalating yet underappreciated public health issue in China [70]. The increase in OC burden appears closely linked to shifts in reproductive behaviors over the past few decades, especially since the implementation of China’s “one-child policy” in 1979, which significantly reduced fertility rates. The well-established “incessant ovulation” and “gonadotropin” hypotheses offer insight into how these reproductive changes may contribute to rising burden of OC [71]. According to the “incessant ovulation” hypothesis, the cumulative number of ovulations increases OC risk, as each ovulatory cycle is believed to cause minor trauma to the ovarian epithelium [72]. With China’s prolonged period of low fertility, this hypothesis suggests that a greater number of ovulations per woman may be contributing to increased OC risk. Similarly, the “gonadotropin” hypothesis proposes that elevated levels of pituitary gonadotropins stimulate the formation of inclusion cysts, which are linked to OC risk [73, 74]. Increased parity, which reduces both ovulatory cycles and gonadotropin exposure, has been shown to decrease OC risk, with each full-term pregnancy lowering risk by over 10% [75–78]. Despite recent relaxations in family planning policies, fertility rates have continued to decline, influenced by economic pressures and shifting societal norms. National trends showed a drop in birth rates from 1990 to 2020 [79], and China’s population experienced a net decrease in 2022 [80], suggesting persistently low fertility intentions. These long-standing shifts in reproductive patterns, therefore, likely play a significant role in the observed increase in OC burden.

Our study observed distinct age-specific trends in OC burden among Chinese women. The ASIR increased to varying degrees between 20 and 94 years, with the age-standardized DALYs rate increasing after 50 years. Notably, both indicators increased the fastest at 70–79 years. The peak ages for ASIR and DALYs were mainly concentrated at 50–55 years. Their peak ages showed a delayed trend, which differed from that of a previous study [81]. Although OC incidence remains lower than BC or CC, it remains highly lethal. More than 70% of cases are diagnosed at advanced stages, owing to a lack of effective screening techniques and the asymptomatic early stage of the disease. These findings underscore the importance of focusing future research on age-specific patterns and the need for improved early detection methods for OC to better understand and manage this high-burden cancer.

We obtained high fasting glucose levels, high BMI, and occupational risk as risk factors associated with DALYs in OC. High fasting glucose levels and diabetes significantly affect the prognosis and survival of patients with OC [82–84]. However, the association between high fasting glucose levels or diabetes and the development of OC remains controversial. Early studies did not find any correlation between them [85–87]. A meta-analysis of 18 studies that controlled for age, BMI, smoking, and alcohol consumption found a significant association between diabetes and OC [88]. A subsequent meta-analysis of 15 cohort studies combining the RR values for type 1 and type 2 diabetes found a strong positive association between both types of diabetes and OC risk. This association was highly significant in Asian population [89]. Moreover, an association between overweight or obesity and the risk of developing OC has been demonstrated [90]. Certain occupational exposures, such as exposure to talc and asbestos in industries, including industrial production, chemical production, printing, and dyeing, may increase the risk of OC [91–93].

### Limitation and Advantages

First, this study was limited by the estimation nature of the GBD2019 data. There may be a discrepancy between the reported and actual data. Second, the different histological subtypes of three cancers may exhibit distinct disease burden trends. However, separate data for the various subtypes are not currently available in the GBD2019 database, which fails to provide a detailed classification of WCs. Third, Variations in diagnostic technologies and changes in screening policies over time were not controlled, which could affect the observed trends in disease burden. The strengths of this study lie mainly in the long-time span of the dataset and the robust methodology.

### Conclusion

This study provides a comprehensive analysis of the trends in incidence and DALYs rates for BC, CC and OC in China from 1990 to 2019. Findings indicate that age-specific patterns and reproductive health policies have significantly influenced the cancer burden among Chinese women. BC and CC exhibited marked growth in ASIR over three decades, with CC showing a slow increase in ASIR due to targeted prevention measures. OC, though less prevalent, demonstrated the highest growth rate in ASIR and age-standardized DALYs rate, underscoring a rising public health concern. These findings emphasize the need for enhanced early screening, targeted education, and tailored risk-reduction strategies for each cancer, particularly addressing age-specific trends to improve women’s health outcomes.

## Data Availability

The disease data used in this study are openly available in GBD 2019 at https://vizhub.healthdata.org/gbd-results/. The population data presented in this study are openly available in the United Nations Population Division’s World Population Prospects (2019 Revision) at https://population.un.org/wpp/Download/Standard/CSV/. Further information is available from the corresponding authors upon request.

## Abbreviations

95% CI, the 95% confidence interval; AAPC, average annual percentage change; APC, the annual percentage change; PAF, the proportion of DALYs attributable to risk factors; ASIR, the age-standardized incidence rate; ASR, Age-standardized rate; BC, breast cancer; CC, cervical cancer; DALYs, disability-adjusted life-years; GBD, the Global Burden of Diseases; HPV, human papillomavirus; OC, ovarian cancer; STIs, sexually transmitted infections; WCs, women’s cancers; WHO, the World Health Organization.
