# Supplementary material for: Trends in the Disease Burden and Risk Factors of Women’s Cancers in China From 1990 to 2019
Source: Int J Public Health. 2024 Dec 4;69:1607245. doi: 10.3389/ijph.2024.1607245 (PMC11652174; doi:10.3389/ijph.2024.1607245)
Supplement: Supplementary file 1 [file DataSheet1.docx]

**Trends in the Disease Burden of Women’s Cancers in China from 1990 to 2019 and Predictions till 2044**

**Supplementary 1: Disease burden of WCs by age in 1990 and 2019 and its time trends from 1990 to 2019**

**Supplementary 2: Detailed results of Age-Period-Cohort Model**

**Supplementary 3: The portion of DALYs of women’s cancers attributable to risk factors**

**Supplementary 1:**

**Disease burden of WCs by age in 1990 and 2019 and its time trends from 1990 to 2019**

**Table S1 The incident cases and age-standardized incidence rates of WCs in 1990 and 2019 and its time trends from 1990 to 2019**

**A. Breast cancer**

|  | 1990 | | 2019 | | 1990-2019 | |
| --- | --- | --- | --- | --- | --- | --- |
| Characteristics | **Incident cases**  **No.** | **ASIR**  **per 100 000** | **Incident cases**  **No.** | **ASIR**  **per 100 000** | **AAPC in ASIR**  **No. (95% CI)** |  |
| **Overall** | | | | | **2.6* (2.5, 2.7)** |  |
| **Age** |  |  |  |  |  |  |
| **15 to 19** | 238 | 0.0332 | 230 | 0.0506 | 1.5* (1.2, 1.7) |  |
| **20 to 24** | 557 | 0.0717 | 757 | 0.1495 | 2.5* (1.7, 3.4) |  |
| **25 to 29** | 1269 | 0.1939 | 3374 | 0.5386 | 3.7* (2.2, 5.3) |  |
| **30 to 34** | 3140 | 0.5631 | 10884 | 1.3431 | 3.0* (1.6, 4.5) |  |
| **35 to 39** | 7794 | 1.2660 | 17057 | 2.5992 | 2.5* (2.1, 2.9) |  |
| **40 to 44** | 9723 | 2.0672 | 30082 | 4.0651 | 2.3* (1.7, 2.9) |  |
| **45 to 49** | 9879 | 2.5605 | 44389 | 4.4358 | 2.0* (1.0, 3.0) |  |
| **50 to 54** | 11305 | 2.8108 | 58297 | 5.2429 | 2.2* (1.8, 2.5) |  |
| **55 to 59** | 11735 | 2.6927 | 52011 | 5.1025 | 2.0* (0.6, 3.5) |  |
| **60 to 64** | 8974 | 2.0746 | 46713 | 4.5291 | 2.7* (2.1, 3.4) |  |
| **65 to 69** | 6858 | 1.5436 | 44317 | 3.6177 | 2.9* (2.1, 3.7) |  |
| **70 to 74** | 4541 | 0.9473 | 27548 | 2.8145 | 3.8* (3.7, 4.0) |  |
| **75 to 79** | 2818 | 0.6277 | 16642 | 1.8443 | 3.7* (3.1, 4.3) |  |
| **80 to 84** | 1524 | 0.4159 | 10370 | 1.0373 | 3.2* (2.4, 3.9) |  |
| **85 to 89** | 568 | 0.2094 | 4121 | 0.3956 | 2.1* (1.2, 2.9) |  |
| **90 to 94** | 127 | 0.0791 | 1266 | 0.1244 | 2.0* (1.5, 2.5) |  |
| **95+** | 23 | 0.0298 | 317 | 0.0322 | 1.0 (-0.1, 2.2) |  |

Notes: ASIR: age-standardized incidence rate; AAPC: average annual percentage changes; CI: confidence interval.

**B. Cervical Cancer**

|  | 1990 | | 2019 | | 1990-2019 | |
| --- | --- | --- | --- | --- | --- | --- |
| Characteristics | **Incident cases**  **No.** | **ASIR**  **per 100 000** | **Incident cases**  **No.** | **ASIR**  **per 100 000** | **AAPC in ASIR**  **No. (95% CI)** |  |
| **Overall** | | | | | **1.1* (0.7, 1.4)** |  |
| **Age** |  |  |  |  |  |  |
| **15 to 19** | 292 | 0.0407 | 182 | 0.0400 | 0.0 (-0.5, 0.4) |  |
| **20 to 24** | 750 | 0.0965 | 580 | 0.1146 | 0.7 (-0.1, 1.5) |  |
| **25 to 29** | 1444 | 0.2206 | 2041 | 0.3258 | 1.2 (-0.7, 3.1) |  |
| **30 to 34** | 2718 | 0.4875 | 6375 | 0.7867 | 1.8* (0.0, 3.5) |  |
| **35 to 39** | 4233 | 0.6876 | 8254 | 1.2578 | 2.1* (1.8, 2.5) |  |
| **40 to 44** | 4296 | 0.9134 | 11567 | 1.5631 | 2.0* (1.6, 2.3) |  |
| **45 to 49** | 4145 | 1.0743 | 14850 | 1.4840 | 1.2* (0.1, 2.4) |  |
| **50 to 54** | 4493 | 1.1171 | 17761 | 1.5973 | 1.3* (0.9, 1.7) |  |
| **55 to 59** | 4997 | 1.1466 | 14078 | 1.3811 | 0.6 (-0.3, 1.6) |  |
| **60 to 64** | 4045 | 0.9351 | 10582 | 1.0260 | 0.3 (-0.6, 1.1) |  |
| **65 to 69** | 3447 | 0.7758 | 9310 | 0.7600 | -0.2 (-0.8, 0.5) |  |
| **70 to 74** | 2793 | 0.5827 | 6315 | 0.6452 | 0.4* (0.2, 0.5) |  |
| **75 to 79** | 1779 | 0.3963 | 3850 | 0.4267 | 0.3 (-0.2, 0.8) |  |
| **80 to 84** | 873 | 0.2382 | 2446 | 0.2447 | 0.2 (-0.1, 0.4) |  |
| **85 to 89** | 314 | 0.1158 | 1168 | 0.1121 | -0.1 (-0.5, 0.3) |  |
| **90 to 94** | 53 | 0.0330 | 329 | 0.0323 | -0.2 (-1.4, 1.1) |  |
| **95+** | 9 | 0.0117 | 73 | 0.0074 | -1.6 (-3.3, 0.1) |  |

**C. Ovarian Cancer**

|  | 1990 | | 2019 | | 1990-2019 | |
| --- | --- | --- | --- | --- | --- | --- |
| Characteristics | **Incident cases**  **No.** | **ASIR**  **per 100 000** | **Incident cases**  **No.** | **ASIR**  **per 100 000** | **AAPC in ASIR**  **No. (95% CI)** |  |
| **Overall** | | | | | **2.0* (1.9, 2.1)** |  |
| **Age** |  |  |  |  |  |  |
| **0-14** | 237 | 0.0381 | 188 | 0.0414 | 0.4 (-0.2, 0.9) |  |
| **15 to 19** | 462 | 0.0644 | 327 | 0.0719 | 0.4* (0.1, 0.6) |  |
| **20 to 24** | 665 | 0.0856 | 635 | 0.1254 | 1.3* (0.9, 1.7) |  |
| **25 to 29** | 619 | 0.0946 | 960 | 0.1532 | 1.7* (0.9, 2.5) |  |
| **30 to 34** | 711 | 0.1275 | 1454 | 0.1794 | 1.0 (-0.1, 2.2) |  |
| **35 to 39** | 1034 | 0.1680 | 1777 | 0.2708 | 1.7* (1.3, 2.0) |  |
| **40 to 44** | 1158 | 0.2462 | 2854 | 0.3857 | 1.5* (0.8, 2.1) |  |
| **45 to 49** | 1176 | 0.3048 | 4716 | 0.4713 | 1.5* (1.0, 2.0) |  |
| **50 to 54** | 1380 | 0.3431 | 6646 | 0.5977 | 2.0* (1.6, 2.3) |  |
| **55 to 59** | 1450 | 0.3327 | 6006 | 0.5892 | 1.9* (1.2, 2.6) |  |
| **60 to 64** | 1186 | 0.2742 | 5517 | 0.5349 | 2.4* (1.9, 2.9) |  |
| **65 to 69** | 987 | 0.2221 | 5747 | 0.4691 | 2.5* (2.3, 2.8) |  |
| **70 to 74** | 754 | 0.1573 | 4069 | 0.4157 | 3.4* (3.3, 3.5) |  |
| **75 to 79** | 479 | 0.1067 | 2243 | 0.2486 | 3.0* (2.7, 3.2) |  |
| **80 to 84** | 254 | 0.0693 | 1461 | 0.1461 | 2.6* (2.3, 3.0) |  |
| **85 to 89** | 104 | 0.0383 | 628 | 0.0603 | 1.6* (0.6, 2.5) |  |
| **90 to 94** | 22 | 0.0137 | 217 | 0.0213 | 1.8* (1.3, 2.3) |  |
| **95+** | 3 | 0.0381 | 37 | 0.0414 | 0.5 (-0.6, 1.5) |  |

**Table S2 The DALYs and age-standardized DALY rates of WCs in 1990 and 2019 and its time trends from 1990 to 2019**

**A. Breast cancer**

|  | 1990 | | 2019 | | 1990-2019 | |
| --- | --- | --- | --- | --- | --- | --- |
| Characteristics | **DALYs**  **No.** | **Age-standardized DALYs rates**  **per 100 000** | **DALYs**  **No.** | **Age-standardized DALYs rates**  **per 100 000** | **AAPC in age-standardized DALYs rates (95% CI)** |  |
| **Overall** | | | | | **-0.2* (-0.3, 0.0)** |  |
| **Age** |  |  |  |  |  |  |
| **15 to 19** | 4557.39 | 0.6348 | 1691.31 | 0.3717 | -1.8* (-2.1, -1.6) |  |
| **20 to 24** | 10855.38 | 1.3969 | 5667.49 | 1.1193 | -0.8 (-1.6, 0.1) |  |
| **25 to 29** | 24274.34 | 3.7084 | 25014.69 | 3.9928 | 0.5 (-0.8, 1.8) |  |
| **30 to 34** | 65012.23 | 11.6594 | 90720.13 | 11.1947 | -0.1 (-1.5, 1.2) |  |
| **35 to 39** | 161998.49 | 26.3135 | 142759.09 | 21.7545 | -0.7* (-0.9, -0.4) |  |
| **40 to 44** | 182326.35 | 38.7650 | 223102.75 | 30.1489 | -0.9* (-1.5, -0.3) |  |
| **45 to 49** | 177444.02 | 45.9908 | 328130.64 | 32.7899 | -1.1* (-2.2, -0.1) |  |
| **50 to 54** | 210641.34 | 52.3724 | 485160.16 | 43.6325 | -0.6* (-0.9, -0.3) |  |
| **55 to 59** | 210455.81 | 48.2917 | 436737.52 | 42.8460 | -0.6 (-2.0, 0.8) |  |
| **60 to 64** | 144240.77 | 33.3453 | 354024.49 | 34.3247 | 0.1 (-0.6, 0.8) |  |
| **65 to 69** | 101874.45 | 22.9292 | 321066.94 | 26.2092 | 0.4 (0.0, 0.9) |  |
| **70 to 74** | 65640.05 | 13.6934 | 211582.03 | 21.6165 | 1.6* (1.4, 1.7) |  |
| **75 to 79** | 38262.61 | 8.5228 | 130215.23 | 14.4307 | 1.8* (1.4, 2.2) |  |
| **80 to 84** | 18203.58 | 4.9675 | 76585.52 | 7.6607 | 1.5* (1.0, 2.1) |  |
| **85 to 89** | 6162.65 | 2.2721 | 31714.12 | 3.0444 | 0.9* (0.1, 1.7) |  |
| **90 to 94** | 1331.00 | 0.8294 | 10985.14 | 1.0798 | 1.3* (0.8, 1.8) |  |
| **95+** | 205.90 | 0.2665 | 2083.11 | 0.2117 | -0.2 (-1.3, 1.0) |  |

Notes: DALYs: Disability adjusted life years.

**B. Cervical Cancer**

|  | 1990 | | 2019 | | 1990-2019 | |
| --- | --- | --- | --- | --- | --- | --- |
| Characteristics | **DALYs**  **No.** | **age‐standardized DALY rates**  **per 100 000** | **DALYs**  **No.** | **age‐standardized DALY rates**  **per 100 000** | **AAPC in age‐standardized DALY rates (95% CI)** |  |
| **Overall** | | | | | **-0.3* (-0.6, -0.1)** |  |
| **Age** |  |  |  |  |  |  |
| **15 to 19** | 5203.68 | 0.7248 | 1706.75 | 0.3751 | -2.2* (-2.4, -2.0) |  |
| **20 to 24** | 13678.02 | 1.7601 | 5542.74 | 1.0947 | -1.6* (-2.3, -0.9) |  |
| **25 to 29** | 26105.31 | 3.9881 | 19491.51 | 3.1112 | -1.0 (-2.9, 0.9) |  |
| **30 to 34** | 46996.29 | 8.4284 | 58117.53 | 7.1716 | -0.4 (-2.1, 1.3) |  |
| **35 to 39** | 80004.68 | 12.9952 | 84358.61 | 12.8551 | 0.0 (-0.4, 0.4) |  |
| **40 to 44** | 91802.94 | 19.5185 | 140158.90 | 18.9403 | 0.0 (-0.8, 0.8) |  |
| **45 to 49** | 97384.14 | 25.2405 | 215682.84 | 21.5531 | -0.4 (-1.6, 0.7) |  |
| **50 to 54** | 109595.22 | 27.2490 | 292770.76 | 26.3301 | -0.1 (-0.3, 0.2) |  |
| **55 to 59** | 118178.81 | 27.1176 | 238968.14 | 23.4439 | -0.5 (-1.4, 0.4) |  |
| **60 to 64** | 91237.44 | 21.0921 | 181390.63 | 17.5868 | -0.7 (-1.4, 0.1) |  |
| **65 to 69** | 73255.76 | 16.4879 | 163499.52 | 13.3467 | -0.8* (-1.5, -0.2) |  |
| **70 to 74** | 54277.76 | 11.3230 | 108726.01 | 11.1081 | -0.1 (-0.2, 0.1) |  |
| **75 to 79** | 30523.38 | 6.7989 | 60653.69 | 6.7217 | 0.0 (-0.3, 0.4) |  |
| **80 to 84** | 12631.80 | 3.4471 | 33215.33 | 3.3225 | -0.1 (-0.3, 0.2) |  |
| **85 to 89** | 3829.06 | 1.4118 | 13795.49 | 1.3243 | -0.2 (-0.6, 0.2) |  |
| **90 to 94** | 566.31 | 0.3529 | 3453.88 | 0.3395 | -0.2 (-1.5, 1.0) |  |
| **95+** | 88.15 | 0.1141 | 709.35 | 0.0721 | -1.7 (-3.5, 0.2) |  |

**C. Ovarian Cancer**

|  | 1990 | | 2019 | | 1990-2019 | |
| --- | --- | --- | --- | --- | --- | --- |
| Characteristics | **DALYs**  **No.** | **Age-standardized DALY rates**  **per 100 000** | **DALYs**  **No.** | **Age-standardized DALY rates**  **per 100 000** | **AAPC in age-standardized DALY rates (95% CI)** |  |
| **Overall** | | | | | **1.3* (1.2, 1.4)** |  |
| **Age** |  |  |  |  |  |  |
| **0-14** | 4869.39 | 0.7822 | 2503.02 | 0.5509 | -1.1* (-1.9, -0.2) |  |
| **15 to 19** | 7386.92 | 1.0289 | 3344.87 | 0.7352 | -1.2* (-1.4, -0.9) |  |
| **20 to 24** | 10944.44 | 1.4084 | 6716.90 | 1.3266 | -0.2 (-0.6, 0.1) |  |
| **25 to 29** | 10277.30 | 1.5701 | 10299.18 | 1.6439 | 0.1 (-0.3, 0.6) |  |
| **30 to 34** | 14367.30 | 2.5767 | 19741.00 | 2.4360 | -0.3 (-1.4, 0.9) |  |
| **35 to 39** | 21724.28 | 3.5287 | 25475.57 | 3.8821 | 0.4* (0.0, 0.7) |  |
| **40 to 44** | 27336.03 | 5.8120 | 47443.07 | 6.4112 | 0.3 (-0.3, 0.8) |  |
| **45 to 49** | 29045.45 | 7.5281 | 87283.03 | 8.7222 | 0.5* (0.0, 1.0) |  |
| **50 to 54** | 36130.32 | 8.9832 | 142121.20 | 12.7816 | 1.3* (0.9, 1.7) |  |
| **55 to 59** | 36154.25 | 8.2960 | 127605.95 | 12.5188 | 1.3* (0.6, 2.0) |  |
| **60 to 64** | 27923.35 | 6.4553 | 114332.37 | 11.0852 | 1.9* (1.4, 2.4) |  |
| **65 to 69** | 21129.63 | 4.7557 | 111262.92 | 9.0826 | 2.2* (1.9, 2.4) |  |
| **70 to 74** | 14629.31 | 3.0519 | 73068.56 | 7.4651 | 3.1* (3.0, 3.3) |  |
| **75 to 79** | 8190.14 | 1.8243 | 35942.21 | 3.9832 | 2.7* (2.4, 3.1) |  |
| **80 to 84** | 3448.68 | 0.9411 | 18175.26 | 1.8180 | 2.3* (2.0, 2.7) |  |
| **85 to 89** | 1253.00 | 0.4620 | 7328.17 | 0.7035 | 1.5* (0.5, 2.4) |  |
| **90 to 94** | 222.01 | 0.1383 | 2088.95 | 0.2053 | 1.7* (1.1, 2.2) |  |
| **95+** | 4869.39 | 0.7822 | 2503.02 | 0.5509 | 0.2 (-0.8, 1.3) |  |

**Supplementary 2:**

**Detailed results of Age-Period-Cohort Model**

**Table S3: The definitions of age-period-cohort model parameters**

| **Effects** | **connotation** | **Parameter** | **Definition** |
| --- | --- | --- | --- |
| **Age effect** | refers to age-related physiological and pathological changes that affect disease mortality rates | The longitudinal age curve | The fitted longitudinal age-specific rates relative to the reference cohorts adjusted for period deviations |
| **Period effect** | refers to changes in disease mortality rate caused by various events over time | The period rate ratios  (period RRs) | the ratios of age-specific rates in a given period compared to the reference period |
| **Cohort effect** | refer to differences in disease mortality rates between generations as a consequence of lifestyle changes over time or different exposure to risk factors | The cohort rate ratios  (cohort RRs) | ratios of age-specific rates in a given cohort compared to the reference cohort |
| Local Drift (% per year) | | | The annual percentage changes in disease burden in expected age-specific rates over time |
| Net Drift (% per year) | | | The annual percentage change in overall disease burden in the expected age-adjusted rates over time |

**Table S4 The results of the local drift values of each age groups and the overall net drift values for the disease burden of WCs in China during 1990-2019.**

|  | **Age** | **Breast Cancer** | | | **Cervical Cancer** | | | **Ovarian Cancer** | | |
| --- | --- | --- | --- | --- | --- | --- | --- | --- | --- | --- |
|  |  | **Incidence**  **(APC, 95% CI)** | **Death**  **(APC, 95% CI)** | **DALYs**  **(APC, 95% CI)** | **Incidence**  **(APC, 95% CI)** | **Death**  **(APC, 95% CI)** | **DALYs**  **(APC, 95% CI)** | **Incidence**  **(APC, 95% CI)** | **Death**  **(APC, 95% CI)** | **DALYs**  **(APC, 95% CI)** |
| **Local**  **Drift** | 22.5 | 2.45  (0.72, 4.22) | -1.57  (-4.46, -1.42) | -1.30  (-3.56, 1.02) | 0.82  (-0.59, 2.25) | -1.94  (-4.13, 0.3) | -1.77  (-3.52, 0.01) | 0.97  (0.45, 1.49) | -0.85  (-1.73, 0.04) | -0.79  (-1.48, -0.1) |
|  | 27.5 | 2.34  (1.52, 3.16) | -1.66  (-2.91, -0.39) | -1.39  (-2.40, -0.36) | 0.85  (0.14, 1.57) | -1.90  (-2.94, -0.86) | -1.73  (-2.59, -0.86) | 0.74  (0.39, 1.1) | -1.07  (-1.63, -0.51) | -0.99  (-1.44, -0.54) |
|  | 32.5 | 2.32  (1.80, 2.84) | -1.56  (-2.28, -0.84) | -1.28  (-1.89, -0.67) | 1.74  (1.26, 2.23) | -0.94  (-1.59, -0.28) | -0.76  (-1.33, -0.19) | 0.77  (0.47, 1.07) | -0.91  (-1.33, -0.49) | -0.84  (-1.2, -0.49) |
|  | 37.5 | 2.05  (1.66, 2.44) | -1.67  (-2.17, -1.17) | -1.40  (-1.84, -0.95) | 2.25  (1.85, 2.65) | -0.22  (-0.71, 0.26) | -0.05  (-0.49, 0.39) | 0.88  (0.63, 1.14) | -0.6  (-0.93, -0.26) | -0.55  (-0.84, -0.26) |
|  | 42.5 | 1.82  (1.51, 2.12) | -1.74  (-2.11, -1.38) | -1.48  (-1.82, -1.14) | 2.37  (2.03, 2.72) | 0.16  (-0.21, 0.52) | 0.31  (-0.04, 0.66) | 1.06  (0.84, 1.28) | -0.22  (-0.46, 0.03) | -0.18  (-0.41, 0.05) |
|  | 47.5 | 1.96  (1.69, 2.23) | -1.50  (-1.80, -1.20) | -1.24  (-1.54, -0.94) | 2.39  (2.05, 2.72) | 0.39  (0.08, 0.7) | 0.53  (0.21, 0.85) | 1.37  (1.17, 1.57) | 0.29  (0.09, 0.49) | 0.33  (0.13, 0.53) |
|  | 52.5 | 2.58  (2.31, 2.85) | -0.83  (-1.11, -0.56) | -0.60  (-0.90, -0.30) | 2.35  (2, 2.71) | 0.65  (0.36, 0.94) | 0.75  (0.43, 1.07) | 2.04  (1.84, 2.23) | 1.17  (0.99, 1.35) | 1.20  (1.01, 1.39) |
|  | 57.5 | 2.75  (2.46, 3.03) | -0.55  (-0.82, -0.28) | -0.37  (-0.68, -0.05) | 1.61  (1.24, 1.98) | 0.24  (-0.05, 0.52) | 0.28  (-0.06, 0.61) | 2.26  (2.06, 2.46) | 1.58  (1.41, 1.75) | 1.58  (1.38, 1.78) |
|  | 62.5 | 3.26  (2.94, 3.57) | 0.16  (-0.12, 0.44) | 0.33  (-0.02, 0.68) | 1.34  (0.94, 1.74) | 0.29  (0, 0.58) | 0.28  (-0.08, 0.65) | 2.69  (2.48, 2.91) | 2.17  (2, 2.35) | 2.18  (1.96, 2.39) |
|  | 67.5 | 3.46  (3.09, 3.83) | 0.59  (0.28, 0.90) | 0.73  (0.31, 1.16) | 0.82  (0.37, 1.27) | 0.06  (-0.24, 0.36) | 0.03  (-0.4, 0.45) | 2.83  (2.58, 3.08) | 2.43  (2.24, 2.61) | 2.44  (2.18, 2.7) |
|  | 72.5 | 3.83  (3.36, 4.31) | 1.25  (0.89, 1.61) | 1.36  (0.81, 1.91) | 0.59  (0.05, 1.13) | 0.11  (-0.23, 0.45) | 0.03  (-0.5, 0.56) | 3.13  (2.83, 3.44) | 2.82  (2.6, 3.05) | 2.84  (2.5, 3.18) |
|  | 77.5 | 4.07  (3.40, 4.75) | 1.78  (1.32, 2.24) | 1.87  (1.09, 2.65) | 0.55  (-0.17, 1.27) | 0.30  (-0.14, 0.73) | 0.18  (-0.56, 0.93) | 3.20  (2.77, 3.62) | 2.95  (2.65, 3.24) | 2.96  (2.46, 3.45) |
|  | 82.5 | 3.96  (2.83, 5.11) | 1.97  (1.24, 2.70) | 2.02  (0.69, 3.36) | 0.70  (-0.47, 1.89) | 0.63  (-0.06, 1.32) | 0.49  (-0.79, 1.79) | 3.07  (2.36, 3.79) | 2.85  (2.37, 3.34) | 2.83  (1.95, 3.73) |
| **Net Drift** | | **2.73***  **(2.52, 2.94)** | **-0.56***  **(-0.80, -0.31)** | **-0.35***  **(-0.59, -0.11)** | **1.58***  **(1.36, 1.80)** | **-0.03**  **(-0.25, 0.19)** | **0.04**  **(-0.19, 0.26)** | **1.90***  **(1.78, 2.03)** | **0.96***  **(0.83, 1.10)** | **0.99***  **(0.86, 1.13)** |

**
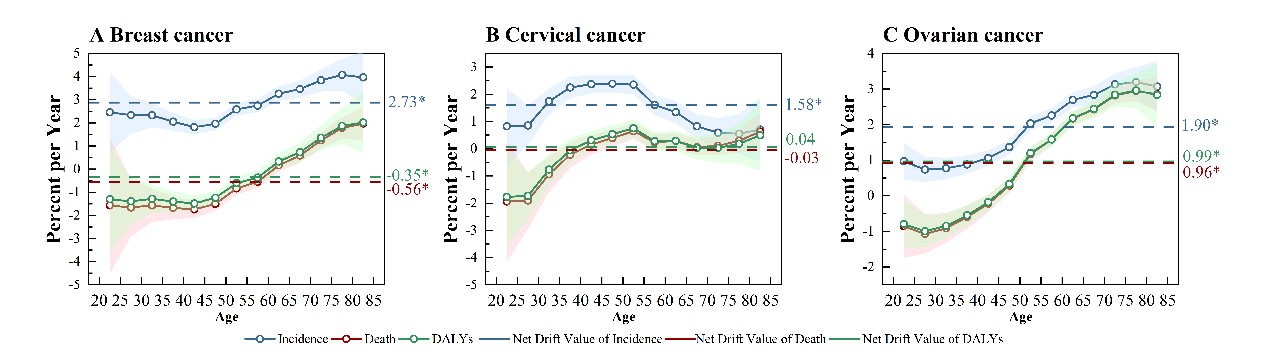
**

**Figure S1 Local drift curves with net drift values of incidence, death, and DALYs for (A) breast cancer, (B) cervical cancer, and (C) ovarian cancer from 1990-2019.**

Figure 1 showed local drift curves and net drift values of disease burden in WCs. Net drift indicates the overall APC of disease burden across the whole study period, whereas local drift indicates the APC in disease burden for each age group relative to the net drift. The overall net drift values were similarly presenting positive for the incidence of breast cancer (2.73*, 95% CI: 2.52, 2.94), cervical cancer (1.58*, 95% CI: 1.36, 1.80), and ovarian cancer (1.90*, 95% CI: 1.78, 2.03), implying the substantial increases in incidence of these three WCs. The increase trend was also striking in the death (0.96*, 95% CI: 0.83, 1.10) and DALYs (0.99*, 95% CI: 0.86, 1.13) of ovarian cancer. Indeed, for death (-0.03, with no statistical significance) and DALYs (0.04, with no statistical significance) of cervical cancer in China, there was no significant trend across the study period, whereas for death (-0.56*, 95% CI: -0.80, -0.31) and DALYs (-0.35*, 95% CI: -0.59, -0.11) of breast cancer, the reduction trend was small but favorable. (Specific results are shown in Table S5)

The variations in disease burden trends across all age groups was revealed by the local drift. Values of incidence lie totally above 0 for each age group of these WCs. The age group of 55 to 85 has a higher APC (2.75%/y to 4.07%/y) of breast cancer incidence than all age group as a whole, and the same is true for ovarian cancer incidence in the age group of 50 to 85 (2.04%/y to 3.20%/y). However, the high growth trend in the incidence of cervical cancer tends to be younger, particularly women between the ages of 30 and 55 (1.74%/y to 2.39%/y). Additionally, patients with ovarian cancer over 45 (death: 0.29%/y to 2.95%/y, DALYs: 0.33%/y to 2.96%/y) or breast cancer over 60 (death: 0.16%/y to 1.97%/y, DALYs: 0.33%/y to 2.02%/y) remained at risk of progressively increasing death and DALYs during the study period. (Specific results are shown in Table S5)

**Supplementary 3:**

**The portion of DALYs of women’s cancers attributable to risk factors**

**Table S5: Trends of portion of DALYs of women’s cancers attributable to risk factors, 1990-2019 (%)**

**A. Breast cancer**

| **Year** | **Alcohol use** | **Dietary risks** | **High BMI** | **High fasting plasma glucose** | **Low physical activity** | **Tobacco** |
| --- | --- | --- | --- | --- | --- | --- |
|  | **PAF (95% UI)** | | | | | |
| **1990** | 2.02(1.51,2.57) | 3.20(1.17,4.39) | 4.38(0.85,10.96) | 3.86(0.71,8.96) | 0.64(0.37,1.14) | 5.14(1.64,8.31) |
| **1991** | 2.04(1.55,2.56) | 3.22(1.19,4.38) | 4.43(0.88,11.00) | 3.97(0.73,9.15) | 0.67(0.37,1.18) | 5.17(1.69,8.34) |
| **1992** | 2.06(1.56,2.59) | 3.25(1.20,4.41) | 4.50(0.91,11.15) | 4.05(0.76,9.32) | 0.69(0.38,1.21) | 5.20(1.70,8.34) |
| **1993** | 2.08(1.57,2.60) | 3.28(1.23,4.44) | 4.59(0.96,11.23) | 4.12(0.77,9.40) | 0.70(0.39,1.23) | 5.22(1.75,8.36) |
| **1994** | 2.07(1.55,2.62) | 3.33(1.26,4.49) | 4.44(0.94,10.81) | 4.05(0.75,9.24) | 0.70(0.39,1.23) | 5.23(1.76,8.37) |
| **1995** | 2.07(1.54,2.61) | 3.37(1.30,4.53) | 4.33(0.93,10.49) | 3.98(0.74,9.11) | 0.70(0.38,1.24) | 5.24(1.76,8.40) |
| **1996** | 2.05(1.54,2.57) | 3.41(1.34,4.55) | 4.41(0.96,10.55) | 3.94(0.72,8.94) | 0.69(0.38,1.22) | 5.25(1.79,8.42) |
| **1997** | 2.02(1.53,2.51) | 3.46(1.37,4.59) | 4.52(1.01,10.58) | 3.89(0.72,8.90) | 0.66(0.38,1.17) | 5.25(1.79,8.42) |
| **1998** | 1.97(1.49,2.44) | 3.52(1.42,4.64) | 4.57(1.05,10.57) | 3.79(0.70,8.66) | 0.64(0.37,1.16) | 5.23(1.81,8.36) |
| **1999** | 1.93(1.46,2.41) | 3.57(1.45,4.69) | 4.79(1.11,11.02) | 3.77(0.69,8.59) | 0.63(0.36,1.15) | 5.22(1.82,8.32) |
| **2000** | 1.91(1.45,2.37) | 3.63(1.49,4.75) | 5.00(1.19,11.51) | 3.77(0.69,8.61) | 0.62(0.36,1.14) | 5.21(1.82,8.28) |
| **2001** | 1.89(1.45,2.34) | 3.69(1.55,4.80) | 5.25(1.26,11.77) | 3.86(0.71,8.79) | 0.62(0.36,1.14) | 5.20(1.84,8.24) |
| **2002** | 1.89(1.43,2.35) | 3.74(1.57,4.87) | 5.86(1.45,13.07) | 4.15(0.77,9.43) | 0.62(0.36,1.14) | 5.20(1.88,8.21) |
| **2003** | 1.88(1.44,2.34) | 3.81(1.61,4.96) | 6.44(1.63,14.29) | 4.46(0.83,10.14) | 0.62(0.36,1.15) | 5.20(1.94,8.20) |
| **2004** | 1.88(1.43,2.34) | 3.89(1.66,5.06) | 6.75(1.74,14.69) | 4.65(0.87,10.65) | 0.61(0.36,1.14) | 5.19(1.95,8.20) |
| **2005** | 1.89(1.44,2.36) | 3.97(1.69,5.18) | 7.06(1.85,15.22) | 4.76(0.89,10.83) | 0.61(0.36,1.15) | 5.18(1.95,8.19) |
| **2006** | 1.91(1.46,2.39) | 4.06(1.77,5.29) | 7.36(1.96,15.78) | 4.80(0.90,10.91) | 0.61(0.36,1.15) | 5.16(1.95,8.17) |
| **2007** | 1.96(1.50,2.43) | 4.16(1.87,5.41) | 7.65(2.06,16.32) | 4.83(0.91,10.94) | 0.62(0.36,1.16) | 5.14(1.94,8.14) |
| **2008** | 2.03(1.57,2.51) | 4.26(1.96,5.56) | 7.88(2.19,16.68) | 4.84(0.91,10.97) | 0.63(0.37,1.16) | 5.12(1.92,8.11) |
| **2009** | 2.08(1.61,2.60) | 4.36(2.05,5.69) | 8.05(2.26,16.88) | 4.86(0.91,11.08) | 0.64(0.37,1.17) | 5.09(1.93,8.08) |
| **2010** | 2.13(1.64,2.68) | 4.44(2.11,5.81) | 8.25(2.36,17.24) | 4.90(0.92,11.13) | 0.65(0.37,1.19) | 5.07(1.91,8.03) |
| **2011** | 2.17(1.66,2.70) | 4.51(2.13,5.91) | 8.51(2.47,17.68) | 4.96(0.93,11.23) | 0.65(0.37,1.19) | 5.05(1.92,7.98) |
| **2012** | 2.20(1.69,2.74) | 4.58(2.2,6.02) | 8.88(2.61,18.35) | 5.04(0.95,11.42) | 0.66(0.37,1.20) | 5.04(1.91,7.92) |
| **2013** | 2.24(1.71,2.81) | 4.64(2.26,6.12) | 9.22(2.74,19.00) | 5.12(0.97,11.61) | 0.66(0.37,1.23) | 5.02(1.90,7.90) |
| **2014** | 2.28(1.74,2.87) | 4.69(2.31,6.21) | 9.61(2.89,19.73) | 5.19(0.98,11.70) | 0.67(0.38,1.23) | 5.00(1.90,7.88) |
| **2015** | 2.32(1.75,2.93) | 4.74(2.31,6.27) | 10.05(3.06,20.39) | 5.23(0.98,11.75) | 0.68(0.38,1.25) | 4.99(1.93,7.85) |
| **2016** | 2.34(1.76,2.98) | 4.77(2.32,6.33) | 10.44(3.21,21.13) | 5.12(0.96,11.54) | 0.69(0.38,1.29) | 4.99(1.95,7.80) |
| **2017** | 2.37(1.76,3.05) | 4.80(2.32,6.38) | 10.82(3.35,21.77) | 4.99(0.92,11.37) | 0.71(0.39,1.34) | 4.98(1.94,7.78) |
| **2018** | 2.40(1.77,3.11) | 4.82(2.32,6.42) | 11.22(3.50,22.49) | 5.02(0.93,11.42) | 0.72(0.39,1.36) | 4.99(1.95,7.78) |
| **2019** | 2.44(1.77,3.18) | 4.84(2.32,6.49) | 11.66(3.63,23.11) | 5.10(0.94,11.57) | 0.73(0.39,1.36) | 5.00(1.97,7.78) |
| **growth rate** | 21.07% | 51.51% | 166.09% | 32.21% | 12.49% | -2.80% |

Note: UI: uncertainty interval

**B. Cervical cancer**

| **Year** | **Unsafe sex** | **Tobacco** |
| --- | --- | --- |
|  | **PAF (95% UI)** | |
| **1990** | 100.00 (100.00,100.00) | 9.61(4.66,16.29) |
| **1991** | 100.00 (100.00,100.00) | 9.75(4.71,16.36) |
| **1992** | 100.00 (100.00,100.00) | 9.85(4.81,16.60) |
| **1993** | 100.00 (100.00,100.00) | 9.90(4.83,16.64) |
| **1994** | 100.00 (100.00,100.00) | 9.55(4.65,16.32) |
| **1995** | 100.00 (100.00,100.00) | 9.21(4.52,15.44) |
| **1996** | 100.00 (100.00,100.00) | 9.03(4.45,15.42) |
| **1997** | 100.00 (100.00,100.00) | 8.79(4.33,14.66) |
| **1998** | 100.00 (100.00,100.00) | 8.48(4.22,14.15) |
| **1999** | 100.00 (100.00,100.00) | 8.28(4.09,13.82) |
| **2000** | 100.00 (100.00,100.00) | 8.15(3.95,13.66) |
| **2001** | 100.00 (100.00,100.00) | 8.07(3.95,13.82) |
| **2002** | 100.00 (100.00,100.00) | 8.28(3.98,14.01) |
| **2003** | 100.00 (100.00,100.00) | 8.47(4.05,14.40) |
| **2004** | 100.00 (100.00,100.00) | 8.43(4.01,14.32) |
| **2005** | 100.00 (100.00,100.00) | 8.40(4.02,14.32) |
| **2006** | 100.00 (100.00,100.00) | 8.40(4.01,14.42) |
| **2007** | 100.00 (100.00,100.00) | 8.40(4.05,14.22) |
| **2008** | 100.00 (100.00,100.00) | 8.40(4.02,14.28) |
| **2009** | 100.00 (100.00,100.00) | 8.40(3.98,14.32) |
| **2010** | 100.00 (100.00,100.00) | 8.40(3.96,14.47) |
| **2011** | 100.00 (100.00,100.00) | 8.45(4.00,14.41) |
| **2012** | 100.00 (100.00,100.00) | 8.53(4.06,14.61) |
| **2013** | 100.00 (100.00,100.00) | 8.61(3.99,14.75) |
| **2014** | 100.00 (100.00,100.00) | 8.70(3.97,14.99) |
| **2015** | 100.00 (100.00,100.00) | 8.77(3.94,15.05) |
| **2016** | 100.00 (100.00,100.00) | 8.82(3.90,15.17) |
| **2017** | 100.00 (100.00,100.00) | 8.87(3.87,15.52) |
| **2018** | 100.00 (100.00,100.00) | 8.93(3.91,15.58) |
| **2019** | 100.00 (100.00,100.00) | 8.98(3.99,15.73) |
| **growth rate** | 0.00% | -6.61% |

**C. Ovarian cancer**

| **Year** | **High BMI** | **High fasting plasma glucose** | **Occupational risks** |
| --- | --- | --- | --- |
|  | **PAF (95% UI)** | | |
| **1990** | 0.86(0.00,2.70) | 3.78(0.71,9.37) | 0.89(0.38,1.84) |
| **1991** | 0.88(0.00,2.74) | 3.89(0.74,9.66) | 0.88(0.39,1.77) |
| **1992** | 0.90(0.00,2.78) | 3.96(0.75,9.85) | 0.86(0.37,1.75) |
| **1993** | 0.92(0.00,2.83) | 4.01(0.77,10.01) | 0.84(0.38,1.68) |
| **1994** | 0.94(0.00,2.88) | 4.02(0.77,9.85) | 0.80(0.36,1.58) |
| **1995** | 0.97(0.00,2.95) | 3.99(0.77,9.87) | 0.76(0.34,1.46) |
| **1996** | 1.00(0.00,3.00) | 3.98(0.77,9.78) | 0.74(0.33,1.40) |
| **1997** | 1.04(0.00,3.09) | 3.96(0.76,9.65) | 0.72(0.34,1.38) |
| **1998** | 1.08(0.00,3.17) | 3.91(0.76,9.50) | 0.70(0.32,1.31) |
| **1999** | 1.12(0.00,3.28) | 3.90(0.75,9.50) | 0.70(0.32,1.29) |
| **2000** | 1.16(0.00,3.38) | 3.94(0.77,9.61) | 0.71(0.33,1.30) |
| **2001** | 1.20(0.00,3.47) | 4.09(0.80,9.94) | 0.73(0.33,1.34) |
| **2002** | 1.25(0.00,3.63) | 4.36(0.86,10.50) | 0.76(0.35,1.40) |
| **2003** | 1.30(0.00,3.76) | 4.66(0.92,11.09) | 0.80(0.37,1.42) |
| **2004** | 1.34(0.00,3.82) | 4.90(0.98,11.63) | 0.81(0.38,1.45) |
| **2005** | 1.38(0.00,3.94) | 5.03(1.01,11.98) | 0.81(0.39,1.43) |
| **2006** | 1.42(0.00,4.05) | 5.09(1.02,12.08) | 0.82(0.37,1.40) |
| **2007** | 1.47(0.00,4.14) | 5.11(1.02,12.12) | 0.83(0.38,1.43) |
| **2008** | 1.52(0.00,4.24) | 5.10(1.01,12.11) | 0.87(0.39,1.50) |
| **2009** | 1.56(0.00,4.32) | 5.11(1.01,12.09) | 0.91(0.39,1.49) |
| **2010** | 1.60(0.00,4.42) | 5.14(1.01,12.17) | 0.94(0.39,1.59) |
| **2011** | 1.64(0.00,4.51) | 5.21(1.02,12.28) | 0.97(0.40,1.64) |
| **2012** | 1.69(0.00,4.59) | 5.29(1.03,12.52) | 0.98(0.39,1.69) |
| **2013** | 1.73(0.00,4.68) | 5.37(1.04,12.71) | 0.98(0.37,1.69) |
| **2014** | 1.77(0.00,4.75) | 5.45(1.04,12.89) | 0.97(0.37,1.70) |
| **2015** | 1.81(0.00,4.83) | 5.49(1.05,13.00) | 0.95(0.37,1.64) |
| **2016** | 1.85(0.00,4.91) | 5.37(1.02,12.77) | 0.94(0.37,1.68) |
| **2017** | 1.89(0.00,4.99) | 5.23(1.00,12.56) | 0.93(0.37,1.62) |
| **2018** | 1.94(0.00,5.10) | 5.25(1.02,12.54) | 0.94(0.37,1.70) |
| **2019** | 1.99(0.00,5.20) | 5.35(1.03,12.75) | 0.94(0.38,1.65) |
| **growth rate** | 130.72% | 41.27% | 6.03% |

**Table S6: DALYs rate and Portion of DALYs of women’s cancers attributable to risk factors for age groups in 2019**

**A. Breast cancer**

| **Year** | **Alcohol use** | | **Dietary risks** | | **High BMI** | | **High fasting plasma glucose** | | **Low physical activity** | | **Tobacco** | |
| --- | --- | --- | --- | --- | --- | --- | --- | --- | --- | --- | --- | --- |
|  | **DALYs rate**  **(per100.000)** | **PAF**  **(%)** | **DALYs rate**  **(per100.000)** | **PAF**  **(%)** | **DALYs rate**  **(per100.000)** | **PAF**  **(%)** | **DALYs rate**  **(per100.000)** | **PAF**  **(%)** | **DALYs rate**  **(per100.000)** | **PAF**  **(%)** | **DALYs rate**  **(per100.000)** | **PAF**  **(%)** |
| **0-19** | 0.03 | 0.53 | 0.00 | 0.00 | 0.00 | 0.00 | 0.00 | 0.00 | 0.00 | 0.00 | 0.00 | 0.00 |
| **20-24** | 0.23 | 1.56 | 0.00 | 0.00 | 0.00 | 0.00 | 0.00 | 0.00 | 0.00 | 0.00 | 0.00 | 0.00 |
| **25-29** | 0.81 | 1.76 | 2.43 | 5.29 | 0.00 | 0.00 | 0.38 | 0.82 | 0.22 | 0.47 | 2.07 | 4.50 |
| **30-34** | 2.71 | 1.91 | 7.53 | 5.29 | 0.00 | 0.00 | 1.72 | 1.21 | 0.67 | 0.48 | 6.43 | 4.53 |
| **35-39** | 6.22 | 2.16 | 15.20 | 5.27 | 0.00 | 0.00 | 4.92 | 1.71 | 1.35 | 0.47 | 13.62 | 4.73 |
| **40-44** | 11.78 | 2.63 | 23.49 | 5.24 | 0.00 | 0.00 | 10.55 | 2.35 | 2.08 | 0.47 | 22.25 | 4.97 |
| **45-49** | 14.37 | 2.61 | 28.24 | 5.13 | 0.00 | 0.00 | 18.29 | 3.32 | 2.63 | 0.48 | 27.37 | 4.97 |
| **50-54** | 20.66 | 2.65 | 39.37 | 5.05 | 136.26 | 17.43 | 35.10 | 4.50 | 4.02 | 0.52 | 41.62 | 5.34 |
| **55-59** | 22.47 | 2.43 | 45.41 | 4.91 | 161.07 | 17.35 | 51.65 | 5.58 | 5.47 | 0.59 | 46.71 | 5.05 |
| **60-64** | 22.66 | 2.51 | 43.50 | 4.81 | 155.22 | 17.09 | 58.59 | 6.47 | 6.44 | 0.71 | 45.63 | 5.04 |
| **65-69** | 20.80 | 2.32 | 41.31 | 4.61 | 147.02 | 16.36 | 63.82 | 7.13 | 7.87 | 0.88 | 43.67 | 4.88 |
| **70-74** | 21.05 | 2.44 | 38.57 | 4.47 | 130.06 | 15.03 | 64.96 | 7.54 | 9.49 | 1.10 | 41.84 | 4.85 |
| **75-79** | 19.11 | 2.31 | 34.87 | 4.21 | 111.98 | 13.47 | 63.61 | 7.68 | 11.43 | 1.38 | 41.78 | 5.05 |
| **80-84** | 15.27 | 2.13 | 26.44 | 3.69 | 75.76 | 10.55 | 54.02 | 7.55 | 14.12 | 1.97 | 35.81 | 5.00 |
| **85-89** | 12.29 | 2.14 | 20.60 | 3.59 | 61.26 | 10.64 | 41.45 | 7.24 | 11.79 | 2.06 | 28.76 | 5.01 |
| **90-94** | 12.99 | 2.20 | 20.88 | 3.53 | 63.44 | 10.70 | 39.91 | 6.76 | 12.54 | 2.12 | 28.62 | 4.84 |
| **95+** | 11.63 | 2.29 | 18.34 | 3.61 | 55.00 | 10.79 | 31.22 | 6.15 | 11.31 | 2.23 | 24.23 | 4.77 |

**B. Cervical cancer**

| **Age group** | **Unsafe sex** | | **Tobacco** | |
| --- | --- | --- | --- | --- |
|  | **DALYs rate**  **(per100.000)** | **PAF**  **(%)** | **DALYs rate**  **(per100.000)** | **PAF**  **(%)** |
| **0-14** | 0.00 | 0.00 | 0.00 | 0.00 |
| **15-19** | 4.87 | 100.00 | 0.00 | 0.00 |
| **20-24** | 14.18 | 100.00 | 0.00 | 0.00 |
| **25-29** | 35.86 | 100.00 | 0.00 | 0.00 |
| **30-34** | 91.09 | 100.00 | 2.64 | 2.89 |
| **35-39** | 170.43 | 100.00 | 5.03 | 2.94 |
| **40-44** | 281.64 | 100.00 | 16.83 | 5.99 |
| **45-49** | 362.29 | 100.00 | 21.56 | 5.95 |
| **50-54** | 470.23 | 100.00 | 40.02 | 8.53 |
| **55-59** | 506.33 | 100.00 | 56.33 | 11.14 |
| **60-64** | 464.01 | 100.00 | 51.36 | 11.07 |
| **65-69** | 456.24 | 100.00 | 59.05 | 12.98 |
| **70-74** | 443.27 | 100.00 | 55.85 | 12.62 |
| **75-79** | 386.09 | 100.00 | 47.65 | 12.38 |
| **80-84** | 310.65 | 100.00 | 35.81 | 11.52 |
| **85-89** | 249.60 | 100.00 | 29.77 | 11.91 |
| **90-94** | 185.93 | 100.00 | 22.85 | 12.29 |
| **95+** | 172.86 | 100.00 | 20.86 | 12.06 |

**C. Ovarian cancer**

| **Age group** | **High BMI** | | **High fasting plasma glucose** | | **Occupational risks** | |
| --- | --- | --- | --- | --- | --- | --- |
|  | **DALYs rate**  **(per100.000)** | **PAF**  **(%)** | **DALYs rate**  **(per100.000)** | **PAF**  **(%)** | **DALYs rate**  **(per100.000)** | **PAF**  **(%)** |
| **0-14** | 0.00 | 0.00 | 0.00 | 0.00 | 0.00 | 0.00 |
| **15-19** | 0.00 | 0.00 | 0.00 | 0.00 | 0.00 | 0.00 |
| **20-24** | 0.16 | 0.91 | 0.00 | 0.00 | 0.00 | 0.00 |
| **25-29** | 0.23 | 1.21 | 0.16 | 0.84 | 0.00 | 0.00 |
| **30-34** | 0.44 | 1.42 | 0.38 | 1.24 | 0.00 | 0.00 |
| **35-39** | 0.85 | 1.65 | 0.89 | 1.74 | 0.01 | 0.00 |
| **40-44** | 1.77 | 1.84 | 2.28 | 2.40 | 0.10 | 0.00 |
| **45-49** | 3.00 | 2.04 | 4.95 | 3.38 | 0.26 | 0.00 |
| **50-54** | 5.10 | 2.23 | 10.47 | 4.60 | 0.71 | 0.31 |
| **55-59** | 5.99 | 2.21 | 15.37 | 5.68 | 1.84 | 0.68 |
| **60-64** | 6.37 | 2.17 | 19.19 | 6.56 | 3.12 | 1.06 |
| **65-69** | 6.41 | 2.06 | 22.40 | 7.22 | 4.47 | 1.43 |
| **70-74** | 5.64 | 1.88 | 22.73 | 7.63 | 6.72 | 2.24 |
| **75-79** | 3.85 | 1.68 | 17.82 | 7.79 | 6.22 | 2.70 |
| **80-84** | 2.22 | 1.30 | 12.99 | 7.66 | 5.22 | 3.05 |
| **85-89** | 1.75 | 1.31 | 9.69 | 7.33 | 4.68 | 3.51 |
| **90-94** | 1.49 | 1.32 | 7.68 | 6.84 | 4.57 | 4.04 |
| **95+** | 1.05 | 1.33 | 4.90 | 6.23 | 4.45 | 5.59 |
